# Preparation of TiO_2_ Nanotube Array on the Pure Titanium Surface by Anodization Method and Its Hydrophilicity

**DOI:** 10.1155/2021/2717921

**Published:** 2021-12-26

**Authors:** Jianguo Lin, Wenhao Cai, Qing Peng, Fanbin Meng, Dechuang Zhang

**Affiliations:** ^1^School of Materials Science and Engineering, Xiangtan University, Xiangtan, 411105 Hunan, China; ^2^Key Laboratory of Advanced Technologies of Materials (Ministry of Education), School of Materials Science and Engineering, Southwest Jiaotong University, Chengdu 610031, China

## Abstract

In this work, a highly ordered TiO_2_ nanotube array on pure titanium (Ti) was prepared by anodization. The effects of the applied voltage and anodization time on the microstructure of the TiO_2_ nanotube arrays were investigated, and their hydrophilicity was evaluated by the water contact angle measurement. It was found that a highly ordered array of TiO_2_ nanotubes can be formed on the surface of pure Ti by anodized under the applied voltage of 20 V and the anodization time in the range of 6-12 h, and the nanotube diameter and length can be regulated by anodization time. The as-prepared TiO_2_ nanotubes were in an amorphous structure. After annealing at 550°C for 3 h, the amorphous TiO_2_ can be transformed to the anatase TiO_2_ through crystallization. The anatase TiO_2_ array exhibited a greatly improved hydrophilicity, depending on the order degree of the array and the diameter of the nanotubes. The sample anodized at 20 V for 12 h and then annealed at 550°C for 3 h exhibited a superhydrophilicity due to its highly ordered anatase TiO_2_ nanotube array with a tube diameter of 103.5 nm.

## 1. Introduction

Titanium (Ti) and its alloys have a broad application prospect as implant materials due to their high specific strength, low elastic modulus, excellent corrosion behaviour, and biocompatibility [1–5]. However, the surface of the Ti alloys will be coated with a cystic fiber membrane if they are directly implanted into the human body due to their bioinert, and thus, it is hard for the bioinert alloys to quickly form firm binding to the surrounding tissue, resulting in loosening or even shedding of the implants [6–8]. Therefore, many efforts have been done to devote to the improvement of bioactivity of Ti alloys through the surface modification of Ti alloys in the past decades [9]. In 2001, Sulka et al. [10] first successfully prepared an array of TiO_2_ nanotubes on the surface of Ti by anodized oxidation in an electrolyte containing hydrofluoric acid. The array is highly ordered with a uniform tube diameter, which can effectively promote the specific surface area and adsorption capacity of the Ti alloy. The surface with the special structure has received great attention, and much work has been done on the impact of the nanotube TiO_2_ array on the biocompatibility of Ti alloys. It has been found that the nanotube TiO_2_ layer can enhance the osseointegration through the improvement of the adhesion of the hydroxyapatite (HAP) coating deposited onto TiO_2_ [11]. Oh et al. [12] also indicated that the cell adhesion could be improved by up to 400% due to the mechanical interlocking between the HAP coating and the nanotube TiO_2_ layer. Moreover, Park et al. [13] reported that the orderly array of TiO_2_ nanotubes on a Ti alloy surface could promote its corrosion resistance in simulated body fluids. So, nanotubes fabricated on implant material surfaces provide great potential in promoting cell adhesion, proliferation, and differentiation. Moreover, nanotubes also offer the possibility of bacterial infection control by loading the tubes with antibacterial agents [14]. However, to establish the optimum nanotopography of nanotubes for favorable cell response, further studies are needed to find the optimum length and diameter of nanotubes for recognition and adherence by the sensing element of a bone cell.

It is well documented that the hydrophily of an implanting material plays an important role in the improvement of osseointegration through inducing the enrichment of calcium and phosphorus ions in the body fluid, accelerating the binding with bone tissue and wound healing [15]. However, the hydrophilic of the TiO_2_ nanotube array and its relationship with the topography of the TiO_2_ nanotube has not been reported yet. Therefore, in the present work, highly ordered TiO_2_ nanotube arrays were fabricated by electrochemically anodized in a mixed solution of glycerol and NH_4_F aqueous electrolyte, and the effects of the applied voltage and time on the topography of the TiO_2_ nanotube array were investigated. Moreover, the hydrophilicity of the TiO_2_ nanopore array was evaluated.

## 2. Materials and Methods

### 2.1. Preparation of TiO_2_ Nanotube Array

Ti plate (99.9% pure) with 1.0 mm thickness was used as a substrate to grow oxide nanotube arrays. Prior to the anodization, the surface was polished using silicon carbide papers (400, 600, 1000, 1500, and 2000 grits). The samples were ultrasonically cleaned in ethanol and acetone for 20 min successively and then dried in a nitrogen stream. After degreasing, the samples were eroded in a solution of 25 vol.% HF+25 vol.% HNO_3_ for 30 s. Finally, the sample was cleaned in deionized water and dried in air. The samples were anodized in an electrolyte of 50 vol.% glycerol solution containing 0.3 mol/L ammonium fluoride (NH_4_F). The anodization voltages were selected to be 10 V, 15 V, and 20 V, and the anodization time was 3 h, 6 h, 12 h, and 18 h, respectively. After anodizing, the samples were carefully cleaned with the deionized water and dried in air, and then, the as-prepared samples were annealed at 450°C, 550°C, and 650°C for 3 h in air with a heating rate of 10°C·min^−1^.

### 2.2. Microstructure and Hydrophilic Property Characterization

The microstructure of the TiO_2_ nanotube array was characterized at 20 kV by an XL30 S-FEG scanning electron microscope (SEM) equipped with energy dispersive X-ray analysis (EDS). The phase constitutions of the nanotubes were identified by using an X-ray diffractometer (Rigaku D/Max-2500VL/PC) employing Cu-K*α* radiation (*λ* = 1.54178 × 10^−9^ nm). The hydrophilic properties of the TiO_2_ nanotube array were evaluated via the measurement of the contact angle of a water droplet on its surface by a Contact Angle Goniometer (p/n 250-F1, USA).

## 3. Results

### 3.1. Microstructure Characterization

**Figure 1:**
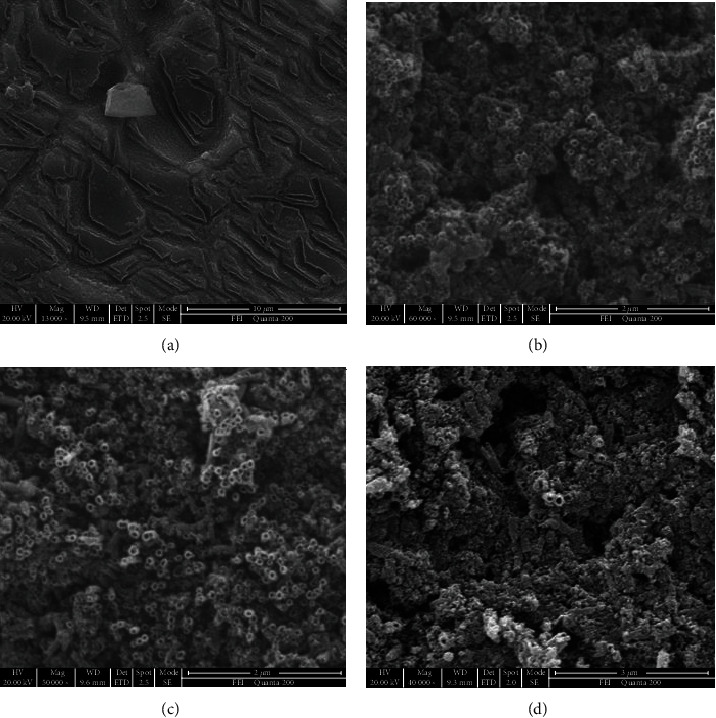
SEM topographies of the TiO_2_ nanotube obtained by different applied voltages: (a) 10 V, (b) 15 V, (c) 20 V, and (d) 40 V.

Figure 1 shows SEM topographies of TiO2 nanotube arrays on the Ti sample anodized under different voltages for 6 h. It is clear that a lot of grooves appeared on the sample surface without the formation of nanotubes after anodized at 10 V (Figure 1(a)). As the voltage increased to 15 V, the regular nanotube array can be observed on the sample surface, and the diameter of the nanotubes was about 40.3 nm (Figure 1(b)). With the voltage further increasing to 20 V, the nanotube array on the sample surface became more regular and ordered, and the average nanotube diameter also was increased to 71.2 nm. However, as the anodization voltage was increased to 40 V, the nanotubes collapsed due to the corrosion of the electrolyte, leading to the order breakdown of the nanotube array.

**Figure 2:**
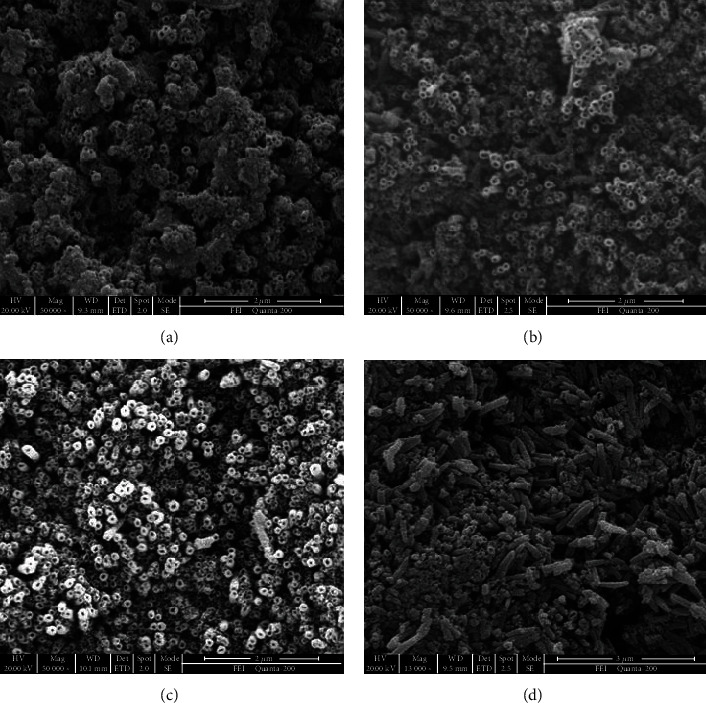
SEM images of the nanotube arrays anodized at 20 V for different times: (a) 3 h, (b) 6 h, (c) 12 h, and (d) 18 h.

To study the effects of the anodization time on the microstructure of the nanotube array, the samples were anodized at 20 V for different times (3 h, 6 h, 12 h, and 18 h) under the constant temperature of 30°C.  Figure 2 are the SEM images showing the surface topographies of the samples after anodized at 20 V for different times. It can be seen that, after being anodized at 20 V for 3 h, the nanotube arrays were formed at some area on the sample surface, but the entire surface of the sample was not completely covered by the nanotube arrays. The average nanotube diameter was about 44.5 nm. With the anodization time extending to 6 h, the regular and ordered nanotube array was formed on the entire surface of the sample, and the average diameter of the nanotubes was increased to about 71.2 nm (Figure 2(b)). With the anodization time further increasing to 12 h, the highly ordered nanotube array with the average nanotube diameter of about 103.5 nm was formed on the entire surface of the sample. However, as the anodization time extended to 18 h, the nanotube in the array began to collapse due to its increased length, resulting in a decrease in array order. Therefore, to obtain a highly ordered array of nanotubes on the surface of pure Ti through anodization in the electrolyte of the present work, the anodization voltage should be selected at 20 V with anodization time ranging from 3 h to 12 h, and the nanotube diameter and length can be regulated in the range of 44.5 nm to 103.5 nm by anodization time.

**Figure 3:**
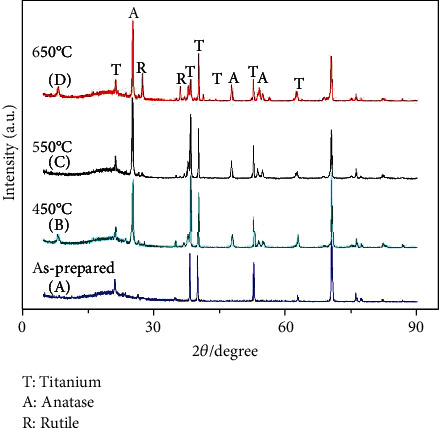
XRD patterns of the nanotube array obtained by anodization at 20 V for 6 h after with annealed at different temperatures: (a) as-prepared, (b) 450°C, (c) 550°C, and (d) 650°C.

To determine the phase structure of the nanotubes, XRD diffraction analysis was performed on the nanotubes array on the surface of the sample anodized at 20 V for 12 h, and the result is shown in Figure 3. It can be seen that the as-prepared nanotubes were in an amorphous structure. To obtain the crystal nanotubes, the sample was annealed at 450°C, 550°C, and 650°C for 3 h, respectively. The XRD patterns of the samples after annealed at different temperatures were also illustrated in Figure 2. It can be seen that the diffraction peaks of the anatase TiO_2_ appeared on the XRD pattern of the sample after annealing at 450°C for 3 h, implying the occurrence of the crystallization from the amorphous nanotubes to the anatase TiO_2_. With the annealing temperature increasing to 550°C, the diffraction peaks of the anatase TiO_2_ became sharper and more intense, indicating that the crystallinity of the nanotubes on the surface of the sample was greatly promoted. As the annealing temperature further increased to 650°C, the diffraction peaks of the rutile TiO_2_ can be observed on the XRD pattern, implying that part of the anatase TiO_2_ transformed into the rutile TiO_2_ during the annealing treatment at 650°C of the nanotube arrays.

### 3.2. Hydrophilicity Property

**Figure 4:**
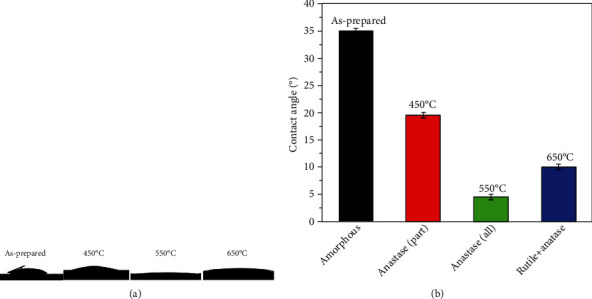
Topographies and contact angles of a water droplet on the surfaces of the TiO_2_ arrays after annealed at different temperatures: (a) topographies and (b) contact angles.

The hydrophilicity of the surface of the anodized samples (anodized at 20 V for 12 h) before and after annealed at different temperatures were investigated by contact angle measurement.  Figure 4 is the images of a water droplet on the surfaces of the samples anodized at different conditions, from which the contact angles of the water droplet with the sample surface can be measured, and the results were illustrated in Figure 3(b). It can be seen that the contact angle of the water droplet with the amorphous TiO_2_ nanotube array surface of the sample anodized at 20 V for 12 h is 23.3°. In contrast, the anodized sample after annealing at 450°C exhibited an improved hydrophilicity due to the formation of the anatase TiO_2_ by partial crystallization of the amorphous nanotubes on its surface, and the contact angle of the water droplet with its surface was decreased to 15.1°. With the annealing temperature increasing to 550°C, the nanotubes were fully crystallized to form the anatase TiO_2_ nanotube array on the sample surface, leading to the further enhancement of its hydrophilicity, and the contact angle of the water droplet with the surface of the sample annealed at 550°C was about 5.0°. However, with the annealing temperature further increasing to 650°C, the rutile TiO_2_ was formed in the nanotube array though the transformation of partial anatase TiO_2_ to rutile TiO_2_, and the hydrophilicity of the surface of the sample annealed at 650°C was decreased slightly and the contact angle of the water droplet with its surface was increased to 9.8°. As a result, the anatase TiO_2_ nanotube array exhibited an excellent hydrophilicity.

**Figure 5:**
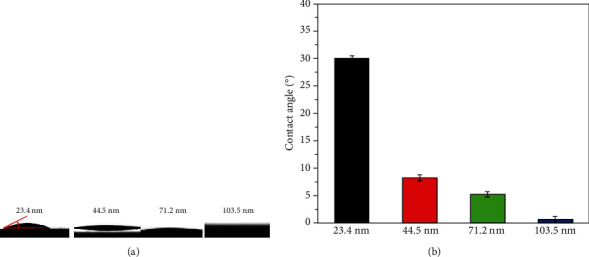
Topographies and contact angles of a water droplet on the surfaces of the TiO_2_ arrays with different tube diameters: (a) topographies and (b) contact angles.

Furthermore, the effects of the nanotube diameter on the hydrophilicity of the anatase TiO_2_ nanotube array were investigated in the present work. To obtain the anatase TiO_2_ nanotube array with different nanotube diameters, the pure Ti samples were firstly anodized at 20 V for 3 h, 6 h, 12 h, and 18 h and then were annealed at 550°C for 3 h, and after that, the anatase TiO_2_ nanotube array with the diameters of 44.5 nm, 71.2 nm, 103.5 nm, and 136.8 nm were obtained, respectively.  Figure 5 showed the contact angles with the surface of with different nanotube diameters. It can be seen that the contact angle on the surface of the anatase TiO_2_ nanotube array on a nanotube diameter of 44.5 nm was about 30.1°, implying its low hydrophilicity. With the increase of the nanotube diameter, the hydrophilicity of the anatase TiO_2_ nanotube array surface increased. As the nanotube diameter increased to 103.5 nm, the anatase TiO_2_ nanotube array surface exhibited superhydrophilicity, and the water contact angle with the surface was close to 0°. However, with the nanotube diameter further increased to 136.8 nm, the hydrophilicity of the TiO_2_ nanotube array decreased due to the collapse of the nanotubes.

## 4. Discussions

It has been documented that the formation of the TiO_2_ nanotube array by electrochemical anodization is the result of the combination of the formation and chemical dissolution of the TiO_2_ barrier layer on the Ti surface under the electric field. At the beginning of the anodization process, a thin TiO_2_ layer (the barrier layer), is quickly formed on the surface of the Ti substrate in the electrolyte. Under the applied electric field, the ions of F^−^ in the electrolyte directly impact the surface of the Ti substrate anode and react with Ti, leading to the formation of a lot of small pits on the Ti surface. In this process, the following reactions are involved in the barrier layer:

(1) $$\begin{array}{c} \text{Ti}+2{\text{H}}_{2}\text{O}-4\text{e}⟶\text{Ti}{\text{O}}_{2}+4{\text{H}}^{+} \end{array}$$

(2) $$\begin{array}{c} {\text{Ti}}^{4+}+6{\text{F}}^{-}⟶{\left[{\text{TiF}}_{6}\right]}^{2-} \end{array}$$

(3) $$\begin{array}{c} {\text{TiO}}_{2}+6{\text{F}}^{-}+4{\text{H}}^{+}⟶\text{Ti}{{\text{F}}_{6}}^{2-}+2{\text{H}}_{2}\text{O} \end{array}$$

Under the electric field, the pits formed by the ions' impact on the Ti substrate surface gradually enlarge and deepen, and the number of the pits per unit area on the barrier layer surface also gradually increases and evenly cover over the surface of the barrier layer to form the original nanopores.

The barrier layer originally formed on the Ti surface is a thin TiO_2_ film with a uniform thickness, and it has the same filed intensity throughout its surface in the applied electric field. However, as the pits are formed on the surface of the barrier layer, the electric field intensity at the bottom of the pit increases, and thus, the Ti-O bond at the pit bottom is weakened under the polarization of the applied electric field, leading to the dissolution of TiO_2_ at this position. In the meaning time, the ions of O^2-^ in the electrolyte move to the Ti substrate/barrier layer interface and react with the Ti matrix to form a new barrier layer at the pit bottom. As a result, the pits in the barrier layer are continuously deepened to form a TiO_2_ nanotube array on the Ti surface.

Therefore, the applied voltage and anodization time have an important impact on the size of TiO_2_ nanotubes and the order degree of the nanotube array. When the applied voltage is too high in the anodization process, a large number of fluorine ions in the electrolyte can obtain greater impact kinetic energy, leading to the formation of a large number of pits with large size high density. These pits overlap each other so that an ordered nanotube array can not form. On the other hand, under the appropriate applied voltage, the anodization time determines the diameter and length of the nanotubes. With the oxidation time increasing, the diameter and length of the nanotubes are due to the corrosion of the electrolyte, but their tube wall is also thinning. So, too long anodization time will make the nanotube walls become too thin, which may cause the nanotubes to collapse. In the case of the present work, a highly ordered array of TiO_2_ nanotubes can be obtained on the Ti surface by the anodization method under the conditions of the applied voltage of 20 V and the anodization time ranging from 3 h to 12 h, and the nanotube diameter can be regulated in the range of 23.4 nm to 103.5 nm by anodization time.

As regards the hydrophilicity of the TiO_2_ nanotube array, it depends on the crystal structure of the TiO_2_, the order degree of the nanotube array, and the tube diameter of the TiO_2_ nanotubes. It was found that the anatase TiO_2_ exhibits the best hydrophilicity in comparison with the amorphous TiO_2_ and the rutile TiO_2_. Thus, after annealing at 550°C for 3 h, the sample with an amorphous TiO_2_ array on its surface exhibited an excellent hydrophilicity due to the transformation of amorphous TiO_2_ to the anatase TiO_2_ through the full crystallization. The results in the present work indicated that the formation of a nanotube array on the Ti surface could significantly improve its hydrophilicity. This is because the nanotube array with a large number of pores can greatly enhance the specific surface area of the Ti surface, where the water droplet can enter the nanotube and the gaps between them, facilitating the smooth paving of the water droplet on the Ti surface.

## 5. Conclusions

In this paper, a highly ordered TiO_2_ nanotube array on pure Ti was prepared by anodization, and their hydrophilicity was evaluated by contact angle of water droplet measurement. The main conclusions are as follows:

1. A highly orderly TiO_2_ nanotube array was successfully prepared on a pure Ti surface by the anodization oxidation method. The applied voltage and anodization time have an important effect on the microstructure of the nanotube array. Under the conditions of the applied voltage of 20 V and the anodization time in the range of 3-12 h, a highly ordered array of TiO_2_ nanotubes can be obtained on the surface of pure Ti, and the nanotube diameter and length can be regulated by anodization time
2. The TiO_2_ nanotubes of the array prepared by anodization were an amorphous structure. After annealing at 550°C for 3 h, the amorphous TiO_2_ array completely transformed to the anatase TiO_2_ array through crystallization. As the annealing temperature increased to 650°C, the partial anatase TiO_2_ nanotubes transformed into rutile TiO_2_ nanotubes
3. The formation of the TiO_2_ nanotubes array on the Ti surface greatly improved its hydrophilicity, which depended on the crystal structure, order degree, and tube diameter of the nanotube array. The sample anodized at 20 V for 12 h and then annealed at 550°C for 3 h exhibited the superhydrophilicity due to its highly ordered anatase TiO_2_ nanotube array with an appropriate nanotube diameter

## Data Availability

The raw/processed data required to reproduce these findings cannot be shared at this time as the data also forms part of an ongoing study.
